# Stress Impact on Resting State Brain Networks

**DOI:** 10.1371/journal.pone.0066500

**Published:** 2013-06-19

**Authors:** José Miguel Soares, Adriana Sampaio, Luís Miguel Ferreira, Nadine Correia Santos, Paulo Marques, Fernanda Marques, Joana Almeida Palha, João José Cerqueira, Nuno Sousa

**Affiliations:** 1 Life and Health Sciences Research Institute (ICVS), School of Health Sciences, University of Minho, Campus Gualtar, Braga, Portugal; 2 ICVS/3B’s - PT Government Associate Laboratory, Braga/Guimarães, Portugal; 3 Clinical Academic Center – Braga, Portugal; 4 Neuropsychophysiology Lab, CIPsi, School of Psychology, University of Minho, Campus Gualtar, Braga, Portugal; University of Maryland, College Park, United States of America

## Abstract

Resting state brain networks (RSNs) are spatially distributed large-scale networks, evidenced by resting state functional magnetic resonance imaging (fMRI) studies. Importantly, RSNs are implicated in several relevant brain functions and present abnormal functional patterns in many neuropsychiatric disorders, for which stress exposure is an established risk factor. Yet, so far, little is known about the effect of stress in the architecture of RSNs, both in resting state conditions or during shift to task performance. Herein we assessed the architecture of the RSNs using functional magnetic resonance imaging (fMRI) in a cohort of participants exposed to prolonged stress (participants that had just finished their long period of preparation for the medical residence selection exam), and respective gender- and age-matched controls (medical students under normal academic activities). Analysis focused on the pattern of activity in resting state conditions and after deactivation. A volumetric estimation of the RSNs was also performed. Data shows that stressed participants displayed greater activation of the default mode (DMN), dorsal attention (DAN), ventral attention (VAN), sensorimotor (SMN), and primary visual (VN) networks than controls. Importantly, stressed participants also evidenced impairments in the deactivation of resting state-networks when compared to controls. These functional changes are paralleled by a constriction of the DMN that is in line with the pattern of brain atrophy observed after stress exposure. These results reveal that stress impacts on activation-deactivation pattern of RSNs, a finding that may underlie stress-induced changes in several dimensions of brain activity.

## Introduction

For many years it has been recognized that acute stress is state of increased vigilance and alertness and to get the organism ready to take action before the impact of dangers [1]. Under brief stressful conditions, the ability to perceive changes in the surrounding environment becomes critical to mount an appropriate response. However, when the homeostatic mechanisms are disrupted, namely through prolonged stress exposure, maladaptive responses take place and trigger inappropriate functional responses with behavioral consequences, including deficits in attention control [2]–[5]. Recently, we showed, both in humans and rodents, that chronic stress triggers long-lasting, but reversible, changes in the frontostriatal networks that govern instrumental behavior decisions with impairments in decision-making processes [6], [7].

It is well established that the brain is organized into multiple spatially distributed large-scale networks; this is evidenced by task-based functional magnetic resonance imaging (fMRI) studies [8]–[10] but also by resting state fMRI studies [11]–[13]. The latter, also known as resting state networks (RSNs), include the default mode (DMN), attention (dorsal and ventral), sensorimotor (SMN), visual (VN), auditory (AN), language and memory networks. The DMN is a network of brain cortical areas that present high metabolic activity when the brain is “at rest” and the individual is not focused on any external demand. This network displays a high degree of functional connectivity between various interacting brain areas. Typically, the DMN comprises areas of the posterior cingulate cortex (pCC) and adjacent precuneus (PCu), the medial prefrontal cortex (mPFC), medial, lateral and inferior parietal cortex, and medial and inferior temporal cortex [14], [15]. The DMN is thought to serve important cognitive functions such as supporting internal mental activity detached from the external world, but also in connecting internal and external attention in monitoring the world around us [16], [17]. There is also evidence that task-induced deactivations of the DMN have been functionally associated with goal-directed behavior [18]. Specifically, deactivation may correspond to a deviation in the default-mode towards a tuning down task-focused behavior that requires attention focus and other demanding cognitive processes. Moreover, task-induced DMN deactivation was related with performance in several cognitive tasks (e.g. [19]), whereas failure of deactivation has been associated with neuropsychiatric diseases (e.g. [20], [21]). While the DMN shows deactivation during cognitively demanding tasks [19], [22], activation in attentional networks (dorsal attention and ventral attention networks) typically increases [23], [24]. Specifically, in addition to the typical DMN, two largely segregated canonical networks in their spatial distribution have also been consistently observed during the brain’s resting state and related with attention-demanding tasks: a bilateral dorsal attention network (DAN), which includes the dorsal frontal and parietal cortices (intraparietal sulcus), and the ventral attention network (VAN), largely right-lateralized, which includes the ventral frontal and parietal cortices (temporo-parietal junction), the insular cortex and subcortical regions [24], [25]. While the DAN has been associated with goal-directed, top-down attention processes as inhibitory control, working memory and response selection, the VAN is related with salience processing and mediates stimulus-driven, bottom-up attention processes [24]–[26]. Moreover, it is relevant to note that dorsal and ventral systems appear to interact not only during cognitive tasks [27], [28] but also during spontaneous activity [25].

In addition to the typical DMN, VAN and DAN, other networks have also been consistently observed during the brain’s resting state, including: the VN involving the occipital and bilateral temporal regions which is linked to the visual processing network and mental imagery [13], [29]; the AN including the superior temporal and inferior frontal gyrus, known for being responsible in auditory processing and language comprehension; the SMN involving the precentral, postcentral gyrus, cerebellum, portion of the frontal gyrus that subserves sensorial and motor tasks [12], [25], [30], [31] and the self-referential network including the medial prefrontal, the anterior cingulate cortex and the hypothalamus [13]. Importantly, all these RSNs were also shown to present abnormal functional patterns in many neuropsychiatric disorders [32]–[36] For example, in autism, RSNs are much more loosely connected [32], [33]; increased functional connectivity was found in social anxiety disorder patients between the right posterior inferior temporal gyrus and the left inferior occipital gyrus, and between the right parahippocampal/hippocampal gyrus and the left middle temporal gyrus [34]; patients with borderline personality disorder showed an increase in functional connectivity in the left frontopolar cortex and the left insula, whereas decreased connectivity was found in the left cuneus [35]; patients with major depressive disorder exhibited increased functional connectivity in the anterior medial cortex regions and decreased functional connectivity in the posterior medial cortex regions compared with controls [36].

Notably, the effect of stress in the functional architecture of RSNs, both during task performance or resting state conditions, is largely unknown. Moreover, although neuropsychiatric diseases (e.g. bipolar disorders, schizophrenia) have been associated with abnormal patterns of RSNs deactivation, which may be related with difficulties in task-focusing and cognitive resources allocation, studies performed during prolonged stress, an established risk factor for neuropsychiatric disorders, are absent. Thus, the main goal of this study was to test if the functional connectivity of RSNs might be aberrant in chronic stress conditions. To achieve this goal, we assessed the architectural differences of RSNs using fMRI independent component analysis (ICA) on resting state data and task induced deactivation analysis, and region-of-interest surface assessments.

## Materials and Methods

### Participants, Psychological Tests and Cortisol Measurements

The participants included in this study were 8 controls (2 males, 6 females; mean age, 24.25±1.98) and 8 stress (2 males, 6 females; mean age, 23.86±0.35) participants submitted to prolonged psychological stress exposure. Control participants included a cohort of medical students under their normal academic activities, whereas the stress group included participants that had just finished their long period of preparation for the medical residence selection exam. Participants responded to a laterality test and to a self-administered questionnaire regarding stress assessment (Perceived Stress Scale – PSS - [37]. Participants were further assessed with the Hamilton anxiety scale [38] and the Hamilton depression scale [39] by a certified psychologist. Upon filling of the questionnaires, and immediately before the MRI and fMRI acquisitions, participants collected saliva samples with the help of Salivette (Sarstedt, Germany) collection devices. Collection took place between 9am and 5pm in all participants. Samples were stored at −20°C until the biologically active, free fraction of the stress hormone cortisol was analyzed using an immunoassay (IBL, Hamburg).

### Ethics Statement

The study was conducted in accordance with the principles expressed in the Declaration of Helsinki and was approved by the Ethics Committee of Hospital de Braga (Portugal). The study goals and tests were explained to all participants and all gave informed written consent.

### Data Acquisition

Participants were scanned on a clinical approved Siemens Magnetom Avanto 1.5 T (Siemens Medical Solutions, Erlangen, Germany) on Hospital de Braga using the Siemens 12-channel receive-only head coil. The different imaging sessions were conducted in the same day and the Siemens Auto Align scout protocol was used to minimize variations in head positioning. For structural analysis and registration to standard space, a T1 high-resolution anatomical sequence, 3D MPRAGE (magnetization prepared rapid gradient echo) was performed with the following scan parameters: repetition time (TR) = 2.4 s, echo time (TE) = 3.62 ms, 160 sagittal slices with no gap, field-of-view (FoV) = 234 mm, flip angle (FA) = 8°, in-plane resolution = 1.2×1.2 mm^2^ and slice thickness = 1.2 mm. During RS-fMRI acquisition, using gradient echo T2* weighted echo-planar images (EPIs), participants were instructed to keep the eyes closed and to think in nothing particular. The imaging parameters were: 100 volumes, TR = 3 s, TE = 50 ms, FA = 90°, in-plane resolution = 3.4×3.4 mm^2^, 30 interleaved slices, slice thickness = 5 mm, imaging matrix 64×64 and FoV = 220 mm. fMRI paradigm acquisition was acquired using: TR = 2 s, TE = 20 ms, FA = 90°, in-plane resolution and slice thickness 3.3 mm, 38 ascending interleaved axial slices with no gap and FoV = 212 mm. The functional paradigm is described in [7] and was presented using the fully integrated fMRI system IFIS-SA.

### Image Pre-processing

Before any data processing and analysis, all the different acquisitions were inspected and confirmed that they were not affected by critical head motion and that participants had no brain lesions.

To achieve signal stabilization and allow participants to adjust to the scanner noise, the first 5 volumes (15 seconds) were discarded. Data preprocessing was performed using SPM8 (Statistical Parametrical Mapping, version 8, http://www.fil.ion.ucl.ac.uk) analysis software. Images were firstly corrected for slice timing using first slice as reference and SPM8’s Fourier phase shift interpolation. To correct for head motion, images were realigned to the mean image with a six-parameter rigid-body spatial transformation and estimation was performed at 0.9 quality, 4 mm separation, 5 mm FWHM smoothing kernel using 2nd degree B-Spline interpolation. No participants exceed head motion higher than 2 mm in translation or 1° in rotation. Images were then spatially normalized to the MNI (Montreal Neurological Institute) standard coordinate system using SPM8 EPI template and trilinear interpolation. Data were then re-sampled to 3×3×3 mm^3^ using sinc interpolation, smoothed to decrease spatial noise with a 8 mm, full-width, half-maximum, Gaussian kernel, temporally band-pass filtered (0.01–0.08 Hz) and the linear trend was removed. The pre-processing of fMRI paradigm images was previously described [7].

### Independent Component Analysis and Identification of RSN

Spatial independent component analysis was conducted for all participants using the Group ICA 2.0d of fMRI Toolbox (GIFT, http://www.icatb.sourceforge.net) [40], [41]. Concisely, ICA analysis consists in extracting the individual spatial independent maps and their related time courses. The reduction of dimensionality of the functional data and computational load was performed with Principal Component Analysis (PCA). 20 components were estimated for each subject and ICA calculation was then performed using the iterative Infomax algorithm. The ICASSO tool was used to assess the ICA reliability, and 20 computational runs were performed on the dataset, during which the components were being recomputed and compared across runs and the robustness of the results was ensured [42]. The independent components were obtained and each voxel of the spatial map was expressed as a t-statistic map, which was finally converted to a z-statistic. Z-statistic describes the voxels that contributed more intensely to a specific independent component, providing a degree of functional connectivity within the network [43], [44]. The final components were visually inspected, sorted and spatially correlated with resting state functional networks from [45]. The best-fit components of each individual (z-maps) were used to perform group statistical analyses.

### RSN Deactivation during fMRI Task Analysis

fMRI paradigm was analyzed by creating a set of regressors at rest and at the time of making a decision, which were convolved with the hemodynamic response function. In order to reliably map task-induced deactivations, we combined all the resting periods (resting baseline condition) and all the decision periods using a protocol previously described [7] (decision condition), given that decision periods were equally demanding. The contrast used to assess task-induced deactivations was the resting baseline condition *minus* decision condition. Resulting functional patterns were masked with the previously described RSNs templates.

### Structural Analysis

Structural analysis based on segmentation of brain structures from T1 high-resolution anatomical data was performed using the freely available Freesurfer toolkit version 5.0 (http://surfer.nmr.mgh.harvard.edu). Intracranial volume (ICV) was used to correct the volumes and the processing pipeline was the same as previously described [7]. DMN was defined by the summed volume of the angular gyrus of inferior parietal lobe, the posterior cingulate, the precuneus and the frontopolar region [14], [15]. The summed volume of the middle frontal gyrus (dorsolateral and prefrontal region) and the posterior parietal region constituted the DAN [46], [47]. VAN was constituted by the sum of the temporal-parietal junction and the ventral frontal cortex volumes [25]. SMN was defined by the summed volume of the paracentral, precentral postcentral and the cerebellum [45]. The summed volume of the cuneus, pericalcarine and the lingual region constituted the primary VN [45].

### Statistical Analyses

Results of the psychological scales, cortisol levels, and regional volumes were analyzed in the IBM SPSS Statistics software, v.19 (IBM, New York). Comparisons between the control and stress groups were done with two-tailed independent-samples t-test. For all these comparisons significance level was set at 0.05. Values are presented as mean ± standard error of the mean.

Group analysis of the fMRI resting state and task induced deactivations were performed using the second level random effect analyses in SPM8. Initially, within group analyses were performed only to confirm the activation of the RS networks in the different groups, using one-sample t-tests. Therefore, between group analyses were implemented with directional two-sample t-tests, to directly compare the groups based on the two experiments designed. Functional results for all RSNs were considered significant at a corrected for multiple comparisons *p*<0.05 threshold (based on the combination of height threshold with a minimum cluster size), determined by Monte Carlo simulation program (AlphaSim). Anatomical labeling was defined by a combination of visual inspection and Anatomical Automatic Labeling atlas (AAL) [48].

## Results

### Physiological and Behavioral Results

**Figure 1:**
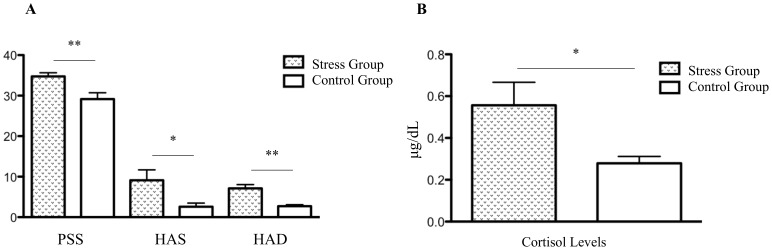
Clinical characteristics of the cohort. **(**A): Perceived Stress Scale (PSS), Hamilton Anxiety Scale (HAS); Hamilton Depression Scale (HAD) and (B): Salivary Cortisol levels of the stressed and control groups. **P<0.01; *P<0.05.

Stress impact was confirmed in several dimensions: in the Perceived Stress Scale (PSS, Figure 1A; *P*<0.007) and in the Hamilton anxiety (HAS, Figure 1A; *P*<0.042) and depression scores (HAD, Figure 1A; *P*<0.001); finally, we found a significant increase in salivary cortisol levels in stressed participants (Figure 1B; *P*<0.042).

### Functional Connectivity Results

**Figure 2:**
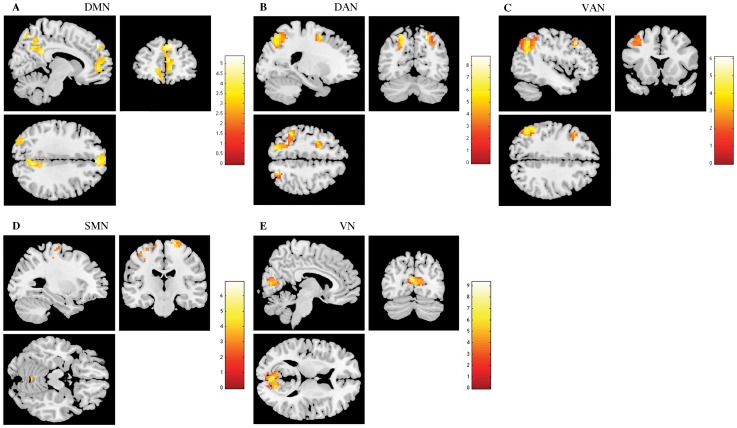
The impact of stress in Resting State Networks (RSNs) at rest. The images depict areas in which stress participants display greater activity than controls in the default mode network (DMN) (A), dorsal attention network (DAN) (B), ventral attention network (VAN) (C), sensorimotor network (SMN) (D) and visual network (VN) (E), extracted by independent component analysis and using two-sample t-tests, with results considered significant at a corrected for multiple comparisons p<0.05 threshold. Noticeable, there were no areas of increased activation of these RSNs in controls than in stress individuals.

The one-sample t-tests revealed a typically spatial pattern of activation (connectivity) and deactivation in DMN, DAN, VAN, SMN, VN and AN in both experimental groups (results not shown). Increased resting functional connectivity was identified in DMN, both attention networks, SMN and VN in the stress group when compared to controls (Figure 2 and Table 1). In contrast, in the comparison control>stress, there was no significant increase of connectivity in any of the studied RSNs. More specifically, in what regards DMN activity, stress increased functional connectivity in the medial prefrontal cortex, medial orbitofrontal cortex, pCC and the precuneus (pCUN) (Table 1). In DAN, increased functional connectivity was found in the superior parietal, right middle occipital and left medial and superior frontal in stress group (Table 1). Increased functional activation was found in the left angular, superior parietal and middle frontal in stressed participants in VAN (Table 1). In SMN, stress increased functional connectivity in the left paracentral lobule, precentral, right postcentral and the left cerebellum (Table 1). Finally, increased functional activation was found in the calcarine in stressed participants in VN (Table 1).

**Table 1:** Group Differences (Stress>Control) at rest, in brain regions of the DMN, DAN, VAN, SMN and VN maps (two sample t-tests, corrected for multiple comparisons, *p*<0.05).

Condition	Regions	Peak MNI coordinates	Cluster size (voxels)	Maximum Z score
Default Mode Network (Stress>Controls)	Frontal superior medial (left)	0, 63, 9	499	3.82
	Frontal medial orbitofrontal (left)	−12, 54, −3		3.64
	Precuneus (right)	3, −63, 39	253	3.78
	Cingulum posterior (right)	9, −45, 30		3.47
	Precuneus (left)	0, −63, 60		3.39
	Occipital middle (left)	−33, −81, 27	61	3.41
Dorsal Attention Network (Stress>Controls)	Parietal superior (right)	21, −66, 51	64	5.03
	Occipital middle (right)	30, −63, 39		4.20
	Frontal superior (left)	−21, 3, 57	71	4.62
	Frontal middle (left)	−30, −3, 51		3.99
	Parietal superior (left)	−18, −72, 48	332	4.61
Ventral Attention Network (Stress>Controls)	Angular (left)	−51, −54, 33	260	4.18
	Parietal superior (left)	−33, −60, 51		2.09
	Frontal middle (left)	−45, 21, 45	70	3.75
Sensorimotor Network (Stress>Controls)	Paracentral Lobule (left)	−15, −27, 69	114	4.49
	Precentral (left)	−18, −12, 78		4.24
	Precentral (right)	18, −27, 69	62	4.11
	Postcentral (right)	33, −30, 54		2.86
	Cerebellum (left)	−3, −60, −18	22	3.42
	Precentral (left)	−42, −12, 51	21	3.27
Visual Network (Stress>Controls)	Calcarine (left)	−3, −72, 15	214	5.18
	Calcarine (right)	15, −72, 12		4.26

**Figure 3:**
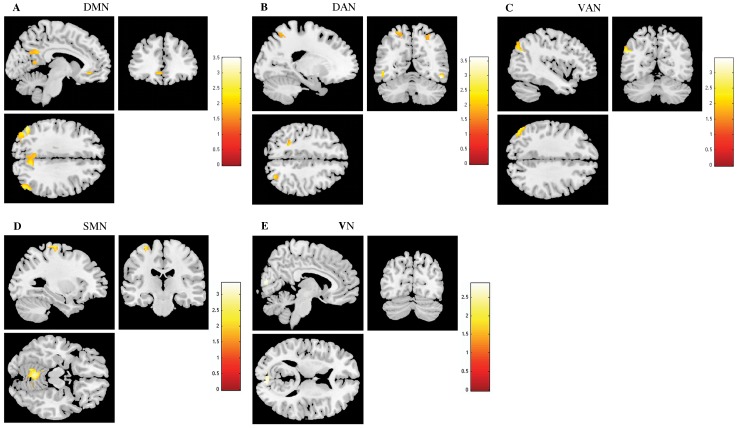
The impact of stress in Resting State Networks (RSNs) during task-induced deactivations. The images illustrate areas of increased deactivation in controls when compared to stressed participants in the default mode network (DMN) (A), dorsal attention network (DAN) (B), ventral attention network (VAN) (C), sensorimotor network (SMN) (D) and visual network (VN) (E), extracted by general linear model analysis and using two-sample t-tests, with results considered significant at a corrected for multiple comparisons p<0.05 threshold. Importantly, no areas of increased deactivation of these RSNs were found in stressed individuals when compared to controls.

In task-induced deactivations, increased deactivations in DMN, both attention networks, SMN and VN were found in controls compared to stressed participants (Figure 3 and Table 2). More specifically, increased deactivations in the left middle occipital, angular and in the pCUN, middle occipital and temporal and parahippocampal right was found in DMN of controls when compared to stress participants (Table 2). In DAN, controls presented higher functional deactivation in the inferior temporal and superior parietal (Table 2). Controls showed an increased deactivation of the VAN, specifically in the left angular and inferior parietal and temporal, compared to stressed participants (Table 2). In SMN, controls presented higher functional deactivation in the cerebellum and in the left precentral (Table 2). Left calcarine was highly deactivated in controls compared to stress participants in the VN (Table 2). No significant region was found to display greater deactivation in stressed participants than in controls in any of the studied RSNs.

**Table 2:** Group Differences (Stress<Control) in brain regions of the DMN, DAN and VAN maps in task-induced deactivation (two sample t-tests, corrected for multiple comparisons, *p*<0.05).

Condition	Regions	Peak MNI coordinates	Cluster size (voxels)	Maximum Z score
Default Mode Network (Stress<Controls)	Occipital middle (left)	−44, −74, 26	282	3.28
	Precuneus (right)	6, −50, 12	747	3.23
	Angular (left)	−50, −68, 30	74	3.01
	Occipital middle (right)	42, −74, 22	351	2.80
	Temporal middle (right)	48, −74, 36		2.59
	Angular (right)	54, −66, 28		2.52
	Parahippocampal (right)	30, −26, −18	86	2.18
Dorsal Attention Network (Stress<Controls)	Temporal inferior (right)	48, −60, −14	60	3.37
	Temporal inferior (left)	−48, −60, −4	61	2.53
	Parietal superior (right)	28, −72, 50	163	2.36
	Parietal superior (left)	−24, −62, 62	115	2.13
Ventral Attention Network (Stress<Controls)	Angular (left)	−48, −68, 30	270	3.24
	Parietal inferior (left)	−28, −80, 44		2.38
	Temporal inferior (left)	−54, −56, −10	73	2.27
Sensorimotor Network (Stress>Controls)	Cerebellum (left)	−6, −56, −12	521	3.16
	Cerebellum (right)	12, −56, −10		2.85
	Precentral (left)	−30, −22, 70	72	2.19
Visual Network (Stress>Controls	Calcarine (left)	4, −86, 14	83	2.74

### Expansion/Contraction Maps of the RSNs

**Figure 4:**
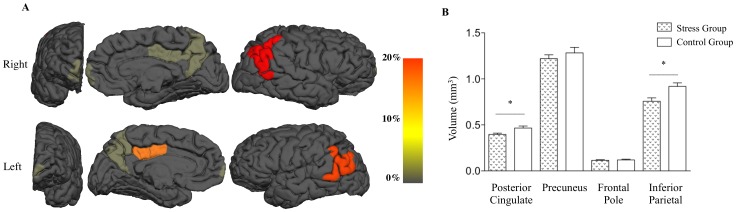
Volumetric Changes in the default mode network (DMN) after stress exposure. (A) Schematic representation of global DMN volumetric changes. (B) Regional volumetric differences in the DMN between Stress and Control groups. *P<0.05.

Whole brain analysis for relative intracranial volumes did not differ between experimental groups. However, a significant reduction (p<0.014) in total DMN volume (corrected ICV) was seen in stressed participants compared to controls (Figure 4). Specific areas of contraction were observed in the left pCC (p<0.025) and the left and right parietal inferior (p<0.024 and p<0.016, respectively). No significant areas of constriction or expansion were found in the dorsal and ventral attention in the SMN and primary VN (p = 0.86, p = 0.55, p = 0.87 and p = 0.67, respectively) between experimental groups.

## Discussion

Herein, we showed for the first time that stress increases the activation of the DMN at rest in the ventral mPFC, pCC, adjacent pCUN and inferior parietal cortex. Based on previous studies highlighting the role of the DMN at rest [16], [17], [49], [50], our results suggest an augment in self-reflective thoughts but also an increased dynamic interaction between emotional processing (i.e., ventral regions) and cognitive functions (i.e., dorsal regions) in stressed participants, as a result of increased activity in the anterior components of the DMN. The increases in the posterior regions of the DMN observed in stressed participants, particularly the pCC and the inferolateral parietal lobes, are likely associated with longer processing of emotionally salient stimuli and episodic memory retrieval [51], [52]. Notably, this increase in functional connectivity was associated with a contraction of the connectivity map of the DMN, with specific reductions in the left pCC and left and right parietal inferior regions. These are likely to reflect the stress-induced atrophic effects in cortical regions, observed in several previous reports [5], [7], although the alternative explanation of a reduction of the number of neurons recruited cannot be completely excluded at this moment.

The characterization of changes in functional connectivity between brain networks subserving distinct psychophysiological functions is of relevance to understand the symptoms triggered by stress, namely mood and anxiety changes [53]. Indeed, stress is well-known to be a precipitating factor for mood changes, a finding confirmed in the present study by the increased scoring in a validated scale of depression. Interestingly, in depressed participants, an increased fMRI connectivity pattern between the “dorsal nexus” (a bilateral dorsal medial prefrontal cortex region) and the DMN has been reported [54]; importantly, this hyperconnectivity has recently been shown to be reduced by antidepressants [55]–[57]. Strikingly, the present study reveals an increased functional connectivity between mPFC (part of dorsal nexus) and pCC in stressed participants. Finally, the finding of increased activation in resting states of the anterior cingulate cortex is also of relevance for the affective processing of negative information, known to be altered in depressed patients [36], [58], [59] but may additionally be related to a higher vigilance and alertness in stressed participants.

Indeed, another behavioral dimension targeted by stress is emotional hyperactivity; again, herein we confirmed that stressed individuals score higher in the Hamilton anxiety scale. The finding of increased connectivity between the superior parietal, right middle occipital and left medial and superior frontal in the stress group suggests that brain regions belonging to DAN could play a role in emotional regulation and in the higher state of vigilance and awareness, which is typical of stressed-induced hyperemotionality. Moreover, the present findings of increased functional connectivity in the pCC in stressed participants than in controls, along with a decreased deactivation in pCUN is of notice. The pCC and the pCUN are sometime referred to as a pivotal hub of the DMN in social cognition and in theory of mind [60]. Our results are also in line with previous fMRI studies revealing a lower deactivation in the pCUN on anxious patients [60], [61]. As a matter of fact, our findings sustain the hypothesis that pCUN would be able to suspend functional connectivity within the DMN, being related to perception of socially relevant emotional state and self-related mental representations [15].

Finally, in accordance with these data evincing emotional hyperactivity as increased anxiety states and related vigilance and alertness, we observed a greater activation of the sensorimotor (SMN), and primary visual (VN) networks in stressed participants. This suggests an hyperactivation of cortical and subcortical attention areas oriented to perception–action, brain systems required to stress-related fight or flight responses. Also, this is in line with disrupted sensorimotor gating mechanisms (necessary to mediate threat selective attention to the most salient signals and ignore other non relevant signals that emerge simultaneously) found in stress conditions [62]. Therefore, our results suggest that stress induces an increase in the general level of alertness and motor response in the stress participants, as suggested by others [63].

Interestingly, the present study reveals not only differences in the pattern of activation of RSNs, but also relevant differences in deactivation of these networks. The VAN was found to be associated with task control function [64]–[66] and to be implicated in “salience” processing [47]. Importantly, the greater functional connectivity found in the VAN during resting state fMRI in stressed participants is likely to be of relevance to understand the decreased functional deactivation of RSNs during task-focused behavior, suggesting a difficulty in moving from more oriented, self-related processes towards a tuning down task-focused behaviour that requires allocation of attention and other cognitive. In fact, it has been shown that the VAN has an important role in cognitive control related to switching between the DMN and task-related networks [46], even thought, the increased rest activity of the DMN in stressed participants might simply require an increased effort for its deactivation during the transition from rest to task-focused activity, which might impact on functional performance. This is consistent with previous studies showing that failure of RSNs deactivation was already evidenced in several neuropsychiatric diseases such as schizophrenia, first-episode psychosis, mild cognitive impairment and mild Alzheimer's disease (e.g. [20], [21], [67]).

Although the RSNs studied herein provide a valuable framework through which alterations of functional connectivity driven by chronic stress exposure can be assessed, they do not cover the whole cortex and thus do not provide a complete description of brain functional architecture. Another limitation of this study relates to the impossibility to provide information on the functional connectivity of RSNs with several regions of the limbic system. In addition, one must still be cautious about the neurophysiological relevance of RSNs, namely on the functional significance of these task-networks when dynamically assembled and modulated during different behavioral states.

In conclusion, while there is substantial evidence for an association between RSNs activation/deactivation abnormalities and psychiatric disorders [68]–[73], this is to the best of our knowledge the first study exploring the functional significance of RSNs patterns after sustained stress exposure. The similarities of present findings with those evidenced by depressed and anxious patients clearly suggest that these patterns of abnormal activity of RSNs in stress participants may represent a neurobiological marker for the stress-induced increased emotionality. The present data, however, also reveals a deficit in the deactivation of the RSNs that reflects an impaired turning off of the un-activated state. Future studies might permit to clarify specific relationship between specific RSNs abnormalities and core phenomena of stress-related disorders as well as whether plastic phenomena also operate after the end of the stress exposure.
